# Purinergic P2 Receptors: Novel Mediators of Mechanotransduction

**DOI:** 10.3389/fphar.2021.671809

**Published:** 2021-05-07

**Authors:** Qihang Kong, Yue Quan, Geer Tian, Junteng Zhou, Xiaojing Liu

**Affiliations:** ^1^ Laboratory of Cardiovascular Diseases, Regenerative Medicine Research Center, West China Hospital, Sichuan University, Chengdu, China; ^2^ Department of Cardiology, West China Hospital, Sichuan University, Chengdu, China

**Keywords:** purinergic P2 receptors, mechanical stress, nucleotides, cellular mechanotransduction, mechanoreceptors

## Abstract

Mechanosensing and mechanotransduction are vital processes in mechanobiology and play critical roles in regulating cellular behavior and fate. There is increasing evidence that purinergic P2 receptors, members of the purinergic family, play a crucial role in cellular mechanotransduction. Thus, information on the specific mechanism of P2 receptor-mediated mechanotransduction would be valuable. In this review, we focus on purinergic P2 receptor signaling pathways and describe in detail the interaction of P2 receptors with other mechanosensitive molecules, including transient receptor potential channels, integrins, caveolae-associated proteins and hemichannels. In addition, we review the activation of purinergic P2 receptors and the role of various P2 receptors in the regulation of various pathophysiological processes induced by mechanical stimuli.

## Introduction

Mechanotransduction refers to the conversion by cells of external mechanical signals into internal biochemical signals (Maurer and Lammerding, 2019). Mechanotransduction enables living organisms to sense a range of mechanical stimuli, including shear stress, stretch-induced strain and osmotic pressure (Wang et al., 2019). Mechanosensors are an important class of molecules that directly and rapidly convert mechanical signals into biochemically relevant signals (Cox et al., 2019). Activation of mechanosensors is the first step in mechanotransduction. There are extensive reviews on mechanotransduction and the ways in which mechanical forces regulate cellular function (Wu et al., 2018; Ozkale et al., 2020). An increasing body of evidence indicates that purinergic P2 receptors (P2Rs) are involved in multiple mechanotransduction pathways.

P2Rs are specialized membrane receptors, which are ubiquitously expressed in various cells and induce biological effects by selectively combining with extracellular nucleotides, such as tri-phosphate nucleosides and di-phosphate nucleosides (Coddou et al., 2011; Di Virgilio et al., 2018). P2Rs encompass a complex network of membrane receptors, including P2X purinergic receptors (P2XRs) and P2Y purinergic receptors (P2YRs). P2XRs are ligand-gated ion channels, while P2YRs are members of seven-membrane-spanning G protein-coupled receptors. Based on differences in their structural and functional characteristics, P2XRs and P2YRs are further subdivided into seven P2X (1–7) subtypes and eight P2Y (1, 2, 4, 6, 11–14) subtypes, respectively (Antonioli et al., 2019).

Adenosine triphosphate (ATP) release and subsequent activation of purinergic receptors are critical in mechanotransduction (Moehring et al., 2018). Cells can directly respond to mechanical stress in the extracellular environment through G protein-coupled receptors and mechanosensitive cation channels (Kefauver et al., 2020; Marullo et al., 2020). In this review, we summarize recent findings on P2R-mediated mechanotransduction and review the role of P2Rs in mechanical force-mediated pathophysiological process.

## Mechanisms of P2RS as Mechanotransducers

The response of cells to mechanical stimuli involves activation of P2Rs and subsequent initiation of intracellular downstream signaling. Elucidation of the mechanisms underlying P2Rs-mediated mechanotransduction could shed light on the optimum approach to target P2Rs in the clinical setting. Multiple mechanosensitive molecules, including transient receptor potential (TRP) channels, integrins, caveolae-associated proteins, hemichannels and piezo1, have been identified that are involved in P2Rs-mediated mechanotransduction.

### Interactions of P2Rs With TRP Channels

The TRP channel family is a type of tetrameric complex located mainly in the plasma membrane, which can be activated directly by a mechanical stimulus (Liu and Montell, 2015; Haustrate et al., 2020). There is evidence supporting mechanical signals inducing interactions between TRP channels and P2Rs, which is mainly reflected in ATP release-mediated coupling of them. For example, in gastrointestinal epithelia, stretch-induced activation of transient receptor potential vanilloid 4 (TRPV4) triggers vesicular nucleotide transporter (VNUT)-mediated ATP exocytosis and increases plasma membrane permeability, resulting in ATP release. ATP release then leads to the activation of P2Rs, and further regulates the production of visceral pain. These findings imply that TRPV4 plays an essential role in P2Rs activation (Mihara et al., 2020). An interaction between transient receptor potential vanilloid 1 (TRPV1) and P2XRs in bladder afferent neurons also appears to be essential for the mechanotransduction during bladder filling (Grundy et al., 2018). Thus, TRPV1 may play a role in mediating bladder mechanosensitivity.

Moreover, in an experiment on cutaneous wound healing, ATP release triggered by mechanical stress activated P2Y2R, which induced long-lasting Ca^2+^ influx into keratinocytes through transient receptor potential canonical 6 (TRPC6) (Takada et al., 2014). In another study, under the action of fluid, endogenously released ATP affected the opening of the TRPV4 and transient receptor potential canonical 1 (TRPC1) by activating P2X4R, thereby regulating the intracellular Ca^2+^ concentration of endothelial cells (Li et al., 2015). Thus, current research strongly suggests that interactions between TRP channels and P2Rs regulate mechanosensory signal transduction. However, as apparent in these studies, P2Rs are not intrinsically mechanosensitive. Whether other interactions between P2Rs and TRP channels exist that do not depend on ATP deserves further study. Studies on the conformation and distribution of P2Rs and TRP channels could shed light on other potential protein–protein interactions.

### Interactions of P2Rs With Integrins

The integrin family forms integrin adhesion sites with various enzymes and membrane-associated proteins located between the extracellular matrix and the cytoskeleton (Osugi et al., 2019). These adhesion sites are essential for integrin-dependent cell adhesion, proliferation, migration and survival (Nolte and Margadant, 2020). A remarkable feature of integrin-mediated adhesion is mechanosensitivity (Sun et al., 2016).

Based on the membrane colocalization of P2Rs and integrins, Cabahug-Zuckerman et al. utilized immunohistochemistry combined with structured illumination super-resolution microscopy for further exploration and then they observed the ‘osteocyte mechanical body’, a specialized mechanotransduction complex, which composed of pannexin 1, P2X7R, T-type Ca^2+^ channel and α_V_β_3_ integrins (Cabahug-Zuckerman et al., 2018). The presence of this specialized mechanotransduction complex may help to explain the remarkable mechanosensitivity of osteocytes. Such unique mechanotransduction mediated by P2X7R and α_V_β3 integrin also appears to be an ATP-based signaling pathway. As P2X7R may mediate the release of ATP (Johnsen et al., 2019), it is necessary to identify the source of ATP. Previous research has also shed light on the role of interactions between P2Y2R and α_V_β_3/5_ integrins in human umbilical vein endothelial cells. Differed from the ATP-based way, the novel spot focused on the Arg-Gly-Asp integrin-binding domain in this research. This domain directly mediates the coupling of P2Y2R and α_V_β_3/5_ integrins. Subsequently, integrin signaling pathways mediated by P2Y2R activate cofilin-1, which modulates shear stress-induced endothelial cytoskeletal alterations, wound closure, and cell alignment (Sathanoori et al., 2017).

As is clear from the above, the interplay between integrins and P2R is vital in maintaining mechanotransduction. Investigations of P2X7R-mediated ATP release are important because both the ligand required for P2X7R and the substance it releases may be ATP. Furthermore, analyses of protein–protein interaction domains of P2Y2R and integrins may reveal potential targets for drug therapy.

### Interaction of P2Rs With Caveolae-Associated Proteins

Caveolae-associated proteins, such as caveolin-1 (Cav-1), caveolin-2 (Cav-2) and caveolin-3 (Cav-3), are essential for the formation and maintenance of the structure of caveolae in cell membranes (Low and Nicholson, 2015). As one of the main raft scaffolding proteins, Cav-1 is involved in mechanotransduction by coupling membrane-bound receptors to downstream signaling molecules (Martinez et al., 2016). Existing evidence points to an important role for Cav-1 in P2R-mediated mechanotransduction. For example, when enterochromaffin cells are mechanically stimulated, Cav-1 associated with cholesterol-rich micro-domains in caveolae forms a scaffold to support the activation of P2Y1R, subsequently leading to the release of 5-hydroxytryptamine (5-HT), which is involved in mucosal secretory reflexes, motility and transmission of information related to visceral pain sensations (Linan-Rico et al., 2016). A recent study using a mechanical injury model system revealed the presence of P2Y2R in Cav-1 raft micro-domains of astrocytoma cells (Martinez et al., 2019). In this study, Cav-1 modulated the cell survival rate by regulating Akt and ERK1/2 phosphorylation, mediated by P2Y2R. The resulting scaffold formed by Cav-1 is essential for P2Y2R signaling. Moreover, P2Y2R and Cav-1 also play a functional role in alveoli via ATP-dependent way, resulting in the regulation of surfactant secretion. Noteworthy, P2Y2R does not exist in the scaffold formed by Cav-1. In this process, in response to mechanical distension, ATP release from alveolar type Ⅰ cells stimulates exocytosis of lamellar bodies of alveolar type Ⅱ cells by increasing the intracellular Ca^2+^ level (Diem et al., 2020).

The interaction of Cav-1 with P2XRs has been previously reported, including Cav-1 acting as a regulatory switch to ensure the efficient activity of P2Rs. In osteoblasts, Cav-1 attenuates P2X7R-mediated mechanotransduction via endocytosis. During this process, Cav-1 detaches from P2X7R and is transported to the cytoplasm (Gangadharan et al., 2015). Additionally, shear stress seems to induce mitochondrial ATP generation of endothelial cells through Cav-1. ATP-mediated P2X4R activation then evokes a Ca^2+^ wave (Yamamoto et al., 2018). In terms of the interaction between Cav-1 and P2Rs, there has been little study on the contact sites or domains between Cav-1 and P2Rs. More research is needed to provide evidence of a direct relation between Cav-1 and P2Rs.

### Functional Interplay of P2Rs With Pannexin and Connexin Hemichannels

There is compelling evidence to support a crucial role of ATP release-mediated coupling of mechanosensitive hemichannels and P2Rs in mechanotransduction. The pannexin family is a class of channel-forming proteins that form large, non-selective plasma membrane channels, which enable the movement of molecules and ions (Chiu et al., 2018; Michalski et al., 2020). The pannexin 1-P2X7R signaling complex is important in mechanotransduction in bone and urinary system (Negoro et al., 2014; Seref-Ferlengez et al., 2016). A pannexin 1-dependent mechanosensitive mechanism modulates ATP signaling and is essential for load-induced skeletal responses and transmission of bladder wall distension signaling (Negoroet al., 2014; Seref-Ferlengez et al., 2019). Under mechanical stretch applied to atrial myocytes, ATP released by pannexin 2 activates P2Rs to induce macrophage migration (Oishi et al., 2012). However, the identity of the specific P2Rs involved in the aforementioned processes remains unknown. In addition, a pannexin 1-dependent mechanosensitive mechanism that modulates P2YRs rather than P2XRs promoted the survival of metastatic cells during the process of intravascular deformation induced by membrane stretch (Furlow et al., 2015).

Connexins, in particular connexin 43, are post-translational phosphorylated proteins involved in mechanotransduction (Plotkin et al., 2015; Delvaeye et al., 2018). P2Rs are known to be involved in connexin channel-dependent mechanotransduction. For instance, in human periodontal ligament cells, continuous compressive forces induce ATP release from connexin 43. During this process, ATP increases the expression and the synthesis of osteopontin and receptor activator of nuclear factor kB ligand by binding to P2Rs (Luckprom et al., 2011). Similarly, in response to mechanical loading, connexin 43-dependent ATP release in chondrocyte accelerated the synthesis of proteoglycan by activating P2Rs (Garcia and Knight, 2010). Coupling of P2Rs and other connexin family members mediated by ATP release has been demonstrated (Svenningsen et al., 2013). In this study, ATP release mediated by connexin 30 and subsequent P2Y2R activation played an important role in regulating renal salt and water reabsorption, maintaining fluid and the electrolyte balance and normal blood pressure. In another study, when bovine corneal endothelial cells underwent severe short-term deformation in response to a local mechanical stimulus, connexin 43-dependent ATP release induced the activation of P2Y1R and P2Y2R on plasma membrane of adjacent cells and subsequent Ca^2+^ wave propagation (Iyyathurai et al., 2016).

As mentioned above, coupling of the hemichannels and P2Rs mediated by ATP release is essential for mechanotransduction. Supplementary Table S1 lists down the key components in the above-mentioned purinergic receptor signaling. Remarkable, the functional characteristics of piezo1 channels are similar to those of hemichannels, as outlined in a recent comprehensive review (Wei et al., 2019). Despite this similarity, the relationship of mechanically activated cation piezo2 with P2Rs has not been reported. Additionally, as a prominent mechanical cue, extracellular matrix stiffness regulates the chondrogenic response of mesenchymal stem cells to hydrostatic pressure by altering P2Rs sensitivity or by some other mechanism that is downstream of P2Rs activation, although the exact type of P2Rs is still unclear (Steward et al., 2016). Based on the current literature on mechanosensor-mediated activation of the purinergic signaling pathway, extracellular mechanical stimuli cannot induce P2R activation directly. Further work is needed to shed light on the effect of mechanical stimuli on P2Rs.

## Involvement of P2Rs in Mechanical Force Mediated Pathophysiological Process

Mechanical forces, such as shear stress, stretch and microgravity, cause physical deformation of cell structure (Cox et al., 2019), which has been a hotspot in cellular biomechanics for decades. Shear stress is the frictional force exerted on cells by a fluid environment (Klems et al., 2020). Mechanical stretch mainly comes from the distension of organs or tissues. These mechanical forces are common in bone and in the gastrointestinal, circulatory and urinary systems (Miyamoto et al., 2014; Kauffenstein et al., 2016; Linan-Rico et al., 2016; Xu et al., 2017). The development and morphogenesis of living organisms require the generation of mechanical forces, which is a critical apart of mechanobiology (Yu et al., 2018).

### P2Rs-Mediated Mechanotransduction in the Cardiovascular System

P2Rs are involved in cardiovascular pathological processes, such as vascular inflammation and atherosclerosis (Wang et al., 2017). As shown in a clinical study, P2Y2R and G_q_/G_11_ (G proteins) control blood pressure by mediating endothelium mechanotransduction (Wang et al., 2015). Another study reported that exposure of endothelium to disturbed blood flow appears to generate multiple inflammatory signals, including p38 signaling, E-selectin and Interleukin-8 secretion, which is regulated by ATP-dependent P2X7R (Green et al., 2018). In this process, P2X7R promotes endothelium inflammation at atheroprone sites by transducing ATP signals into p38 activation. According to the literature, P2X7R might represent a potential therapy for improving endothelial dysfunction and subsequently atherosclerosis. Based on a study on cardiomyocytes, purinergic signaling appears to be related to mechanical stress-induced cardiac fibrosis (Nishida et al., 2008). In this study, pannexin 1-dependent P2Y6R activation in cardiomyocytes induced the expression of fibrogenic factors, which activated cardiac fibroblasts in a paracrine manner. Additionally, in rat atrial myocytes, an increase in Ca^2+^ influx induced by shear stress during a hemodynamic disturbance was abolished by pharmacological blockade of P2Y1R. Thus, it can be seen that P2Y1R plays an important role in regulation of cardiac contraction (Kim and Woo, 2015).

### Regulation of Bone Mechanotransduction by P2Rs

The mechanical environment in which bone cells are embedded is a dynamic combination of various mechanical stimuli, including shear stress, strain and osmotic pressure (Qin et al., 2020). Like in the cardiovascular system, the shear stress of fluid flow is the main force stimulation applied to osteocytes in bone. The abundance of P2Rs in bone points to their potential role in bone homeostasis (Ottensmeyer et al., 2018).

In a murine calvarial osteoblast MC3T3-E1 cell line, when compressive force was applied to MC3T3-E1 cells by centrifugation, the pressure increased extracellular ATP via induction of VNUT (Inoue et al., 2020). Subsequently, VNUT-mediated ATP release inhibited osteoblast differentiation through P2X7R and/or P2Y2R. A recent study also reported that P2Y14R, the only P2YR stimulated by uridine diphosphate sugars, contributed to the differentiation of osteoblasts under a mechanical stimulus, which was confirmed in primary murine osteoblasts and a C2C12-BMP2 osteoblastic cell line (Mikolajewicz and Komarova, 2020). Another study indicated that ATP release induced by low-intensity pulsed ultrasound promotes osteoblast proliferation and osteogenic responses via the activation of P2X7R (Manaka et al., 2015). In addition, P2Y13R contributes to mediation of osteogenic responses to mechanical loading by regulating ATP metabolism in osteoblasts (Wang et al., 2013). Based on a micro-CT analysis of osteoclasts from P2Y2R^−/−^ mice, activation of P2Y2R under mechanical loading regulates bone resorption and mineralization by enhancing ATP release (Orriss et al., 2017).

### Roles of P2Rs in Other Pathophysiological Processes

P2Rs are activated in other organs and systems, such as the gastrointestinal system and bladder. For example, endogenous ATP released after abnormal intestinal wall extension induces visceral pain by activating P2X2R and P2X3R on nociceptive neurons (Wynn et al., 2004). In the process of mucosal stroking reflex of the guinea pig distal colon, 5-HT activates intrinsic primary afferent neurons, and ATP release activates P2Y1R on secretomotor cholinergic neurons, leading to an increase in short circuit current/chloride ion secretion (Cooke et al., 2004). Unlike the aforementioned model, in the touch/stretch reflex of the distal colon of rats, 5-HT release caused activation of P2Y1R, P2Y2R and P2Y4R, resulting in a net increase in chloride secretion (Christofi et al., 2004). P2Y1R also seems to play a major role in increasing 5-HT levels in response to a mechanical stimulus of the ileum mucosal epithelium of the guinea pig (Patel, 2014). Interestingly, in this research, the P2Rs inhibitor had no impact on 5-HT levels in the distal colon. This finding is likely due to differences in interactions between 5-HT and P2Rs in different regions of the gastrointestinal tract. Elucidating the underlying mechanism will benefit our understanding of gastrointestinal tract signaling. In addition, a study has shown that P2X3R plays an important role in colon mechanotransduction and hypersensitivity (Shinoda et al., 2009). However, the specific mechanism of action of P2X3R remains to be confirmed.

During bladder filling, ATP release and subsequent P2YRs activation mediates the storage capacity of the urinary bladder in response to membrane stretch, indicating the importance of P2YRs to the physiological function of the bladder (Mansfield and Hughes, 2014). When urothelial cells are stretched, ATP-dependent P2Rs activation leads to the release of Ca^2+^, thereby preventing an overactive bladder and fecal incontinence (Guan et al., 2018). In mice, the absence of P2Y6R in dorsal root ganglia leads to an increase in the afferent sensitivity of traction stimulation signals, resulting in frequent micturition and a decrease in bladder capacity (Kira et al., 2017).

From a functionality point of view, these literatures point to a key role for P2Rs as mechanotransducers in multiple organs and systems. Supplementary Figure S1 illustrates a proposed relationship between P2Rs and mechanosensitive molecules, and P2Rs mediated-pathophysiological process. Antagonists or agonists of P2YRs are widely used to treat various diseases, such as dry eye syndrome and inflammation (Jacobson et al., 2020). However, the lack of availability of selective agonists for P2XRs remains a problem (Illes et al., 2021). Moreover, given that most drugs still being tested in preclinical experiments, rigorous clinical investigations and subsequent development of new medications targeting P2Rs are required.

## Conclusion

In response to extracellular mechanical stimulus, the interaction of P2Rs with TRP channels, integrins, Cav-1 or hemichannels initiates complex signaling cascades, contributing to changes in the pathophysiological state. Therefore, targeting P2Rs, offers great promise as a novel therapeutic strategy in dysregulated mechanotransduction. Significant advancements have deepened our understanding of the structure and functions of P2Rs in mechanotransduction. Activation of P2Rs seems to be a downstream event caused by the release of nucleotides exposed to a mechanical stimulus. The potential function of P2Rs as direct mechanosensors needs further research. Fortunately, the widespread availability of single-molecule localization microscopy in the area of sub-cellular structures means it is now possible to study interactions between membrane channel proteins and their accessory subunits (Stone et al., 2017). Such studies hold promise in shedding light on the study of the interaction between P2Rs and mechanosensitive molecules.

Future directions for research include studies on:1) the potential role of mechanical stress in direct activation P2Rs, independently of chemical agonists; 2) the protein–protein interaction domains between P2Rs and mechanosensors; 3) ATP release-mediated coupling of the mechanosensitive ion channel piezo2 and P2Rs; 4) the relationship between extracellular matrix stiffness and P2Rs; 5) the role of other P2Rs in mechanotransduction, such as P2X6R, P2Y11R and P2Y12R; 6) and the development and testing of drugs that target P2Rs in mechanotransduction.
