# Supplementary material for: Purinergic P2 Receptors: Novel Mediators of Mechanotransduction
Source: Front Pharmacol. 2021 May 7;12:671809. doi: 10.3389/fphar.2021.671809 (PMC8138185; doi:10.3389/fphar.2021.671809)
Supplement: Supplementary file 1 [file DataSheet2.docx]

Supplementary Table

## Supplementary Table 1

TABLE 1| The key components in P2Rs-mediated mechanotransduction

| Mechanical stimulus | Receptor | Ligands | Cell type | Signaling event | Potential role | Reference |
| --- | --- | --- | --- | --- | --- | --- |
| Stretch | P2Rs | ATP | Gastrointestinal epithelia | TRPV4/VNUT/ATP/P2Rs | Visceral pain | (Mihara et al., 2020) |
| Stretch | P2XRs | ATP | urothelial cells/neurones | TRPV1/ATP/P2XRs | Afferent nerve activity | (Grundy et al., 2018) |
| Stretch | P2Y2R | ATP | Keratinocytes | ATP/P2Y2R/Gq/PLC/DAG/TRPC6/Ca^2+^ influx | Accelerate wound closure | (Takada et al., 2014) |
| Fluid shear stress | P2X4R, P2YRs | ATP | Vascular endothelial cells | ATP/P2X4R, P2YRs/TRPV4-C1/Ca^2+^ influx | Shear stress-induced Ca^2+^ signaling | (Li et al., 2015) |
| Fluid flow | P2X7R | ATP | Osteocytes | α_V_β_3/_ATP/P2X7R/CaV3.2–1/Ca^2+^ influx | Remarkable mechanosensitivity | (Cabahug-Zuckerman et al., 2018) |
| Fluid shear stress | P2Y2R | ATP | Human umbilical vein endothelial cell | ATP/P2Y2R/α_V_β_3/5_/FAK | Cytoskeleton reorganization | (Sathanoori et al., 2017) |
| Mechanical stress | P2Y1R | NTP | Enterochromaffin cells | Cav-1 scaffold/ P2Y1/Gαq/PLC/PIP2/IP3/IP_3_R/ Ca^2+^/ 5-HT | Gut reflexes | (Linan-Rico et al., 2016) |
| Mechanical injury | P2Y2R | ATP/UTP | 1321N1 astrocytoma cells | Cav-1 scaffold /P2Y2R/ Akt, ERK1/2 | Cell survival rate | (Martinez et al., 2019) |
| Stretch | P2Y2R | ATP | Alveolar epithelial type I cells | Cav-1 scaffold/Ca^2+^ influx/pannexin1/ATP/P2Y2R/ | Surfactant secretion | (Diem et al., 2020) |
| Mechanical loading | P2X7R | ATP | MC3T3-E1 cells | ATP/ Cav-1 scaffold/P2X7R | Mechanotransduction signaling cascade | (Gangadharan et al., 2015) |
| Shear stress | P2X4R | ATP | Vascular endothelial cells | ATP/Cav-1/P2X4R/ Ca^2+^ influx | Mechanotransduction signaling cascade | (Yamamoto et al., 2018) |
| Controlled pressure | P2X7R | ATP | Urothelial cell | Pannexin 1/ATP/P2X7R | Urothelial mechanotransduction and signaling | (Negoro et al., 2014) |
| Fluid shear stress | P2X7R | ATP | Osteocytes | Pannexin 1/ATP/P2X7R | Bone mechanotransduction and signaling | (Seref-Ferlengez et al., 2016) |
| Stretch | P2Rs | ATP | Atrial myocytes | Pannexin 2/ATP/P2Rs | Macrophage recruitment | (Oishi et al., 2012) |
| Stretch | P2YRs | ATP | Microvascular metastatic cell | Pannexin 1/ATP/P2YRs | Cell survival | (Furlow et al., 2015) |
| Compressive forces | P2Rs | ATP | Human periodontal ligament cells | Connexin 43/ATP/P2Rs | Expression and the synthesis of osteopontin | (Luckprom et al., 2011) |
| Cyclic loading | P2XRs, P2YRs | ATP | Chondrocytes | Connexin 43/ATP/P2XRs, P2YRs/ Ca^2+^ signaling | Chondrocyte m  echanotransduction pathway | (Garcia and Knight 2010) |
| Fluid shear stress | P2Y2R | ATP | Nephron-collecting duct cells | Connexin 30/ATP/P2Y2Rs/ Ca^2+^ influx | Collecting duct salt and water  reabsorption | (Svenningsen et al., 2013) |
| Mechanical stimulation | P2Y1R, P2Y2R | ATP | Corneal endothelial cells | Connexin 43/ATP/P2Y1R, P2Y2R/ Ca^2+^ signaling | Ca^2+^ wave propagation | (Iyyathurai et al., 2016) |

Mihara H, Boudaka A, Tominaga M, Sugiyama T. (2020). Transient Receptor Potential Vanilloid 4 Regulation of Adenosine Triphosphate Release by the Adenosine Triphosphate Transporter Vesicular Nucleotide Transporter, a Novel Therapeutic Target for Gastrointestinal Baroreception and Chronic Inflammation. Digestion.101:6-11.doi:10.1159/000504021.

Grundy L, Daly DM, Chapple C, Grundy D, Chess-Williams R. (2018). TRPV1 enhances the afferent response to P2X receptor activation in the mouse urinary bladder. Sci Rep. Jan 9;8:197.doi:10.1038/s41598-017-18136-w.

Takada H, Furuya K, Sokabe M. (2014). Mechanosensitive ATP release from hemichannels and Ca(2)(+) influx through TRPC6 accelerate wound closure in keratinocytes. J Cell Sci. Oct 1;127:4159-4171.doi:10.1242/jcs.147314.

Li LF, Xiang C, Qin KR. (2015). Modeling of TRPV(4)-C(1) -mediated calcium signaling in vascular endothelial cells induced by fluid shear stress and ATP. Biomech Model Mechanobiol. Oct;14:979-993.doi:10.1007/s10237-015-0647-3.

Cabahug-Zuckerman P, Stout RF, Jr., Majeska RJ, Thi MM, Spray DC, Weinbaum S, Schaffler MB. (2018). Potential role for a specialized beta3 integrin-based structure on osteocyte processes in bone mechanosensation. J Orthop Res. Feb;36:642-652.doi:10.1002/jor.23792.

Sathanoori R, Bryl-Gorecka P, Muller CE, Erb L, Weisman GA, Olde B, Erlinge D. (2017). P2Y2 receptor modulates shear stress-induced cell alignment and actin stress fibers in human umbilical vein endothelial cells. Cell Mol Life Sci. Feb;74:731-746.doi:10.1007/s00018-016-2365-0.

Linan-Rico A, Ochoa-Cortes F, Beyder A, Soghomonyan S, Zuleta-Alarcon A, Coppola V, Christofi FL. (2016). Mechanosensory Signaling in Enterochromaffin Cells and 5-HT Release: Potential Implications for Gut Inflammation. Front Neurosci.10:564.doi:10.3389/fnins.2016.00564.

Martinez M, Martinez NA, Miranda JD, Maldonado HM, Silva Ortiz WI. (2019). Caveolin-1 Regulates P2Y2 Receptor Signaling during Mechanical Injury in Human 1321N1 Astrocytoma. Biomolecules. Oct 18;9.doi:10.3390/biom9100622.

Diem K, Fauler M, Fois G, Hellmann A, Winokurow N, Schumacher S, Kranz C, Frick M. (2020). Mechanical stretch activates piezo1 in caveolae of alveolar type I cells to trigger ATP release and paracrine stimulation of surfactant secretion from alveolar type II cells. FASEB J. Sep;34:12785-12804.doi:10.1096/fj.202000613RRR.

Gangadharan V, Nohe A, Caplan J, Czymmek K, Duncan RL. (2015). Caveolin-1 regulates P2X7 receptor signaling in osteoblasts. Am J Physiol Cell Physiol. Jan 1;308:C41-50.doi:10.1152/ajpcell.00037.2014.

Yamamoto K, Imamura H, Ando J. (2018). Shear stress augments mitochondrial ATP generation that triggers ATP release and Ca(2+) signaling in vascular endothelial cells. Am J Physiol Heart Circ Physiol. Nov 1;315:H1477-H1485.doi:10.1152/ajpheart.00204.2018.

Negoro H, Urban-Maldonado M, Liou LS, Spray DC, Thi MM, Suadicani SO. (2014). Pannexin 1 channels play essential roles in urothelial mechanotransduction and intercellular signaling. PLoS One.9:e106269.doi:10.1371/journal.pone.0106269.

Seref-Ferlengez Z, Maung S, Schaffler MB, Spray DC, Suadicani SO, Thi MM. (2016). P2X7R-Panx1 Complex Impairs Bone Mechanosignaling under High Glucose Levels Associated with Type-1 Diabetes. PLoS One.11:e0155107.doi:10.1371/journal.pone.0155107.

Oishi S, Sasano T, Tateishi Y, Tamura N, Isobe M, Furukawa T. (2012). Stretch of Atrial Myocytes Stimulates Recruitment of Macrophages via ATP Released Through Gap-Junction Channels. Journal of Pharmacological Sciences. Dec;120:296-304.doi:10.1254/jphs.12202FP.

Furlow PW, Zhang S, Soong TD, Halberg N, Goodarzi H, Mangrum C, Wu YG, Elemento O, Tavazoie SF. (2015). Mechanosensitive pannexin-1 channels mediate microvascular metastatic cell survival. Nat Cell Biol. Jul;17:943-952.doi:10.1038/ncb3194.

Luckprom P, Kanjanamekanant K, Pavasant P. (2011). Role of connexin43 hemichannels in mechanical stress-induced ATP release in human periodontal ligament cells. J Periodontal Res. Oct;46:607-615.doi:10.1111/j.1600-0765.2011.01379.x.

Garcia M, Knight MM. (2010). Cyclic Loading Opens Hemichannels to Release ATP as Part of a Chondrocyte Mechanotransduction Pathway. Journal of Orthopaedic Research. Apr;28:510-515.doi:10.1002/jor.21025.

Svenningsen P, Burford JL, Peti-Peterdi J. (2013). ATP Releasing Connexin 30 Hemichannels Mediate Flow-Induced Calcium Signaling in the Collecting Duct. Frontiers in Physiology.4.doi:ARTN 292

10.3389/fphys.2013.00292.

Iyyathurai J, Himpens B, Bultynck G, D'Hondt C. (2016). Calcium Wave Propagation Triggered by Local Mechanical Stimulation as a Method for Studying Gap Junctions and Hemichannels. Methods Mol Biol.1437:203-211.doi:10.1007/978-1-4939-3664-9_15.
