# Supplementary material for: Purinergic P2 Receptors: Novel Mediators of Mechanotransduction
Source: Front Pharmacol. 2021 May 7;12:671809. doi: 10.3389/fphar.2021.671809 (PMC8138185; doi:10.3389/fphar.2021.671809)
Supplement: Supplementary file 2 [file DataSheet1.docx]

Supplementary Figure

##
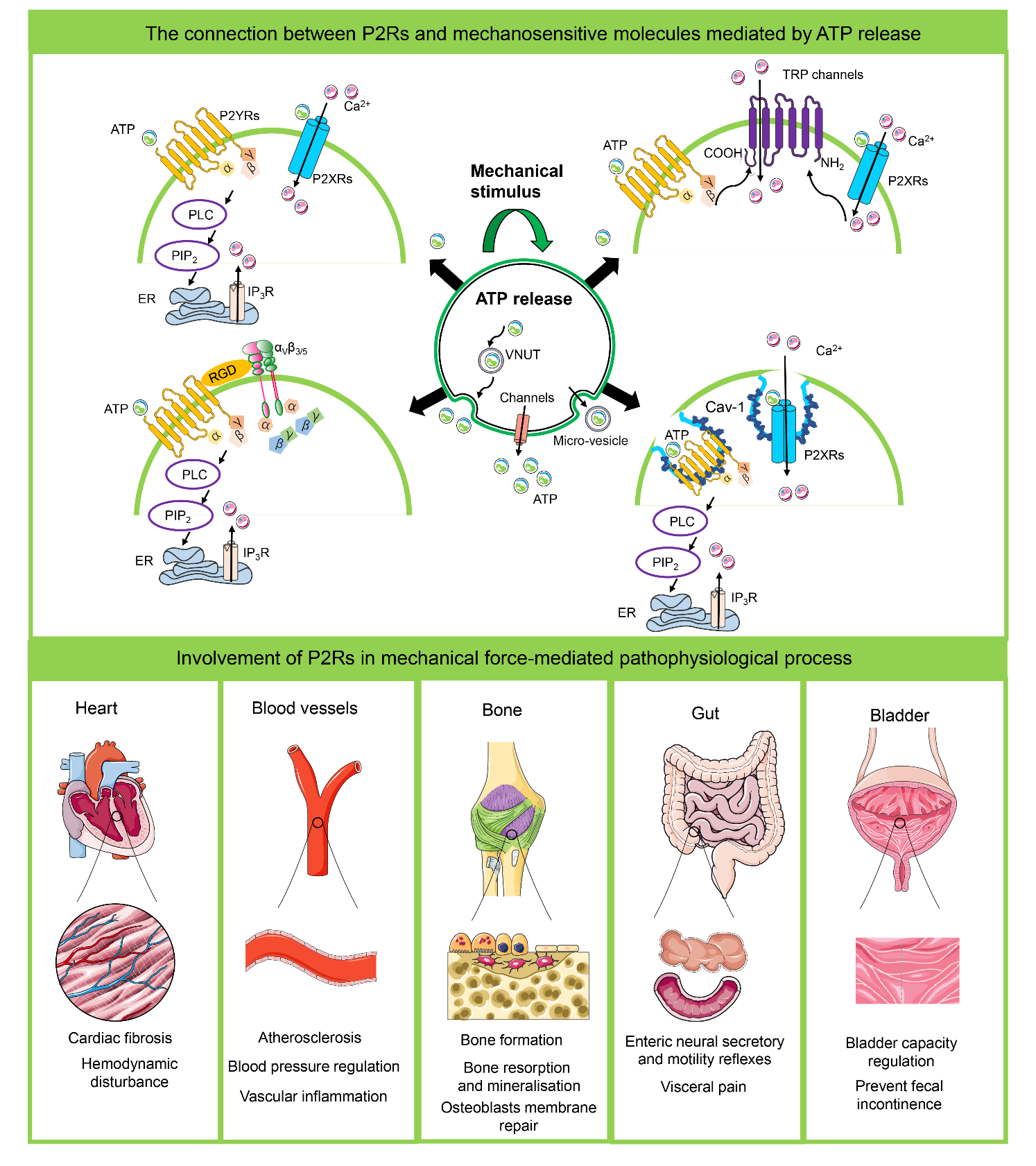


**B**

**A**

## Figure legends

**Figure 1** | A schematic diagram of the proposed P2R-associated purinergic signalling pathway in mechanotransduction. The main mechanisms include ATP release, P2Rs activation and downstream signalling. (A) Intracellular ATP is first released to the extracellular milieu via VNUT-mediated ATP exocytosis, plasma membrane-derived microvesicles or channels (pannexin, connexin or piezo1). Extracellular ATP functions as a key autocrine/paracrine signalling molecule and regulates the Ca2+ concentration and downstream pathway of P2Rs by altering P2R sensitivity. In this process, P2Rs cooperate with mechaosensitive molecules, such as TRP channels, integrins, Cav-1, pannexins and connexins, to regulate mechanotransduction. Abbreviations: RGD, Arg-Gly-Asp integrin-binding domain; ER, endoplasmic reticulum; PLC, phospholipase C; PIP_2_, phosphatidylinositol (4,5) bisphosphate; IP_3_R, 1,4,5-trisphosphate receptors. (B) Involvement of P2Rs in mechanical force-mediated pathophysiological process.
